# Effects of an Overdose of Belladonna

**Published:** 1881-02

**Authors:** 


### EFFECTS OF AN OVERDOSE OF BELLADONNA.

A physician writes to the *Medical Times and Gazette:* —

I once, by mistake, took about two or three grains of the extract of belladonna, and within about half an hour experienced a burning glow over the whole body, with exhileration of spirits, and a feeling of protrusion of the eyeballs. The throat felt intolerably parched, and the tongue felt swollen, and articulation was indistinct. Within an hour a rash appeared universally over the body — a rash which it would be impossible to distinguish from that of scarlatina; the face was bloated, and the hands were swollen. The pupils were not much affected, which I attribute to a large dose of tincture of opium I took soon after the belladonna.

